# Supplementary material for: Primary care networks as a means of supporting primary care: findings from qualitative case study-based evaluation in the English NHS
Source: BMJ Open. 2023 Nov 21;13(11):e075111. doi: 10.1136/bmjopen-2023-075111 (PMC10668191; doi:10.1136/bmjopen-2023-075111)
Supplement: Supplementary data [file bmjopen-2023-075111supp001.pdf]

16.04.20 – v1.2 – IRAS 267050

MANCHESTER 1824 The University of Manchester

Study title: ***Primary Care Networks: exploring primary care commissioning, contracting, and provision***

Topic guide for WP3 interview

- Across which domains have GP practices been collaborating?
- What factors have supported and enabled the development of network working?
- What issues have arisen in developing networks?
- How are networks working with other organisations at ‘neighbourhood’, ‘place’, and ‘system’ levels?
- How have networks attempted to incorporate patient views into decision making about how networks are being organised?
- How have patterns of staff employment in primary care changed as a result of network collaborative working?
- How has network collaborative working contributed to integrated working within neighbourhoods and/or at other scales within ICSs systems?
- Has the operation of PCNs led to any unexpected consequences?
- What was the role of your PCN in the responses to Covid-19 in your local area?
- What factors affected the extent and nature of this role? Have developments changed your view of PCNs and, if so, how?
- How did the Covid-19 emergency influence the development and operation of your PCN? (e.g. changes to the balance of roles recruited for the Additional Roles Reimbursement Scheme?)
- In what ways has Covid-19 affected your PCN’s performance and outcomes?

<u>New Care Models</u>

[…any awareness of the vanguard prog and specifically the EHCH model and the impact of covid on bring things forward]

- How dealing with COVID-19 shaped the development of the EHCH specifications and the operation of PCNs locally?
- Does the new service for your local Care Homes build upon any previous pilots or collaborative work?
- Are you aware of the previous Vanguard programme locally? Are you aware of it nationally?
- Can you point to anything about your new care home service locally that you think has built upon the local Vanguard programme?
- What is helping and hindering the roll out of EHCH model?

<u>Continuity</u>

- The impact on patient experience of continuity as a result of:
    - changes in skill mix within practices (specifically pharmacists and social prescribing link workers);
    - practices working at scale;
    - extended hours services
- Finding the right balance between increasing access for patients and potential resultant decrease in continuity experienced
- How all of the above might differ for different patient groups (i.e. what is most important for older people with polypharmacy, adults with anxiety/depression, working age adults?)
